# Sensorimotor Simulation’s Influence on Stress: EEG and Autonomic Responses in Digital Interviews

**DOI:** 10.3390/brainsci14060608

**Published:** 2024-06-15

**Authors:** Michela Balconi, Laura Angioletti, Katia Rovelli

**Affiliations:** 1 International Research Center for Cognitive Applied Neuroscience (IrcCAN), Università Cattolica del Sacro Cuore, 20123 Milan, Italy; michela.balconi@unicatt.it (M.B.);; 2 Research Unit in Affective and Social Neuroscience, Department of Psychology, Università Cattolica del Sacro Cuore, 20123 Milan, Italy

**Keywords:** stress management, sensorimotor simulation, EEG, autonomic indices, Social Stress Test, stressful interview, cognitive regulation effort, emotional involvement

## Abstract

This study explored the role of sensorimotor simulation in modulating the stress response in individuals exposed to stressful digital simulated interviews. Participants were assigned to two different versions of a Digital Social Stress Test: a simulated version with a dynamic–realistic examining committee (Dyn-DSST) and a version with a static examining committee (Stat-DSST). During interview preparation, behavioral indices reflecting stress regulation and resistance, response times, and electroencephalographic (EEG) and autonomic indices were collected. Higher regulation scores were found for the Stat-DSST group compared to the Dyn-DSST group, probably induced by the presence of limited external sensory input in time and space, perceived as less stressful. The EEG results revealed a distinct contribution of the low- and high-frequency bands for both groups. Dyn-DSST required greater cognitive regulation effort due to the presence of a continuous flow of information, which can enhance sensory and motor activation in the brain. The SCR increased in the Dyn-DSST group compared to the Stat-DSST group, reflecting greater emotional involvement in the Dyn-DSST group and reduced sensory stimulation in the static version. In conclusion, the results suggest that sensorimotor simulation impacts the stress response differently in dynamic interviews compared to static ones, with distinct profiles based on behavioral, EEG, and autonomic measures.

## 1. Introduction

The ability to work under stress and maintain optimal performance under pressure has been deemed a valuable marker of professional maturity and adaptability, as well as subject-specific emotional resilience, which, according to former authors, is essential to successfully face professional challenges and mitigate the risk of errors [1].

In recent years, a significant transformation has occurred in the means by which academic or job interviews take place. Advancements in recruitment have become possible through deep digitalization processes, which have pervaded every aspect of the personnel selection process. In addition, modern organizations are increasingly recognizing that personnel who are capable of proactively regulating and resisting stress are often better able to maintain a consistent level of performance over time.

Stress regulation, defined as the ability to effectively manage physiological and psychological reactions to stress, maintaining a suitable balance in the medium term, plays a pivotal role in fostering neurocognitive efficiency [2,3]. Neurocognitive efficiency can be defined as the brain’s ability to perform optimally in functions such as attention, memory, and executive tasks, particularly under stress. It is characterized by effective information processing and stress regulation, enabling task completion with minimal errors and cognitive effort, and can be assessed through behavioral and neurophysiological measures (e.g., reaction times (RTs), electroencephalographic (EEG) frequency bands) [4,5]. It is particularly crucial when facing tasks or situations under time or psychological pressure. Stress regulation involves an awareness of one’s emotional responses, as well as the ability to cope with challenges, without experiencing prolonged negative impacts on one’s mental and physical health [6]. Moreover, people with greater stress management abilities exhibit a heightened capacity to cope with challenges and sustain optimal cognitive functioning, fostering a constructive mindset under uncertainty, with personal awareness and self-control becoming key aspects [7].

Simultaneously, stress resistance, defined as the capacity to respond promptly and appropriately to more acute and intense stress conditions, further contributes to an individual’s adaptability and performance under pressure [8,9]. It involves not only emotional and mental resilience but also readiness to address situations requiring a rapid and accurate response. Stress resistance implies the ability to maintain high performance in critical situations. In line with the literature, personal awareness [10] and self-control [11] are key components of this capacity, enabling individuals to overcome acute stressors and contribute to a more resilient organizational culture [12]. Therefore, exploring stress regulation and resistance during preparation for a job interview may provide valuable insights into candidates’ abilities to effectively regulate stress and address professional challenges.

Nonetheless, in this context, technology has radically reshaped the conventional job assessment process, transforming the traditional job interview into a virtual experience and, in some cases, an entirely remote experience [13]. Digital simulations, however, can significantly influence the individual’s sensorimotor experience [14], thus affecting their behavioral and neurophysiological responses to stress.

Specifically, as highlighted by previous studies [15,16], sensorimotor integration refers to the process by which sensory information is combined with motor actions, enabling coordinated and adaptive responses to environmental stimuli.

In the context of stress regulation and resilience, this integration is of paramount importance for several reasons. Firstly, sensorimotor integration and simulation play a pivotal role in how individuals perceive and respond to stressors [17]. When a person encounters a stressful situation, such as a demanding digital interview, their sensory systems (e.g., visual and auditory) rapidly process environmental cues [18]. These sensory inputs are then integrated with motor responses, which encompass not only physical actions but also electrophysiological [19,20] and autonomic nervous system responses (e.g., changes in heart rate (HR) and skin conductance) [21]. Research has highlighted the significance of sensorimotor integration in various contexts, including movement disorders, speech motor learning, and stress regulation [22,23,24].

Specifically, sensorimotor simulation, in virtual job interviews, as opposed to those conducted in person, distinguishes itself not only by the peculiarities inherent in interacting with the digital environment, which may restrict sensory perception [25], but also by eliciting a certain emotional detachment, leading to a distinct emotional and cognitive response compared to real-life situations [26].

In general, the literature focusing on the neuroscientific analysis of the differences between face-to-face and remote interactive dynamics is extensive. Recent neuroscience studies have investigated how individuals react to face-to-face (FTF) versus remote computer-mediated (RCM) interview conditions [27]. The primary objective of these studies was to explore and clarify the impacts of the FTF vs. RCM modes on social dynamics within assessment interviews, specifically focusing on interpersonal relationships and inter-brain neural and psychophysiological synchronization in the recruiter–candidate dyad [28,29]. From a neurophysiological standpoint, these studies highlighted higher indices (i.e., intra-brain and inter-brain connectivity indices, computed by the authors using the partial correlation coefficient Πij) in the electroencephalographic (EEG) delta and theta frequency bands in the FTF compared to the RCM condition, regardless of the role (i.e., recruiter or candidate). Regarding autonomic activity, an increase in the skin conductance level (SCL) was found in the FTF condition, interpreted as the arousal reaction to being directly exposed to judgment [28,29].

The functional meaning and scalp localization of the EEG frequency bands can provide insightful information about individuals’ reactions to static versus dynamic conditions. A variation in slower EEG components may indicate greater attention to social processing and the regulation of interpersonal interaction and its affective correlates during the FTF condition compared to the RCM condition [28]. Previous studies have also emphasized the role of theta activity at the scalp level as a contributor to emotional regulation and internal attention and the processing of emotional information [30].

The literature shows that theta is activated by cognitive reward information and, in general, by negative or positive feedback, such as that provided by an examining committee [31]. In addition, alpha band desynchronization has been associated with the modulation of attention processes [32]. Finally, an increase in beta band power in parieto-occipital regions was linked to focused attention [33].

Similarly, the analysis of the autonomous nervous system’s activity can provide valuable insights into an individual’s peripheral reactions during a job interview. It serves as a representative measure of processes related to stress management, self-regulation, and social skills in various contexts, including organizational and professional spheres [34,35]. Notably, the aforementioned studies that have investigated psychological and physiological arousal in this context [28,29], particularly concerning the stress response, focused on the two components of electrodermal activity (EDA): both the slow-varying tonic activity, commonly referred to as the skin conductance level (SCL), associated with arousal and the mental workload [36], and the phasic component, namely the skin conductance response (SCR), reflecting brief changes and serving as an indicator for the evaluation of emotional involvement and the significance attributed to various stimuli [37].

Such literature provides a significant overview of the neurophysiological and autonomic differences that exist between the FTF and RCM dynamics, highlighting how the adoption of a neuroscientific multimethod and multilevel approach, combining EEG frequency bands and autonomic data, has facilitated the investigation of the distinct brain-and-body responses of individuals under FTF and RCM conditions [27]. However, none of these studies have focused on entirely digital environments and the “medium” as a moderator of stress response regulation. The administration of digital interviews and assessments is now the preferred route for many international organizational entities. The digitization of these processes yields numerous benefits, including assessment standardization; nevertheless, digital assessments may induce a modification in the reciprocal directionality between interlocutors. Specifically, sensorimotor simulation in digital job assessments takes on specific prerogatives depending on whether it is conducted in a wholly static condition or in dynamic conditions, where an individual/group X is recorded and individual Y observes the appearance of individual/group X on the screen [38].

Moreover, exploring the neural correlates of stress regulation in digital communication environments holds significance in comprehending the adaptive mechanisms behind human interaction in the digital era and in enhancing the design of digital platforms for communication and assessment objectives. Given these premises, in this study, a sample of healthy adults was divided into two groups, each subjected to a modified version of the Trier Social Stress Test (TSST; [39]), named the Social Stress Test (SST), specifically designed to assess individuals’ behavioral, autonomic, and EEG stress responses to interview-style presentations conducted in two different modalities (static vs. dynamic digital stressful interview).

Former works have demonstrated that participants’ EEG frequency bands reflect their individual responses to the TSST, with a significant increase observed in the delta band during the TSST, considered as mental effort with a negative feeling [40]. Conversely, a decrease was noted for the beta band and, to a lesser extent, for the theta and gamma oscillations, particularly in the frontal region, interpreted as indicating that a negative mood overcomes mental activity and places pressure on a person [40]. However, to the best of our knowledge, no previous studies have compared individuals’ behavioral and neuro- and psycho-physiological responses to modified versions of the TSST when delivered through a different medium.

In the current study, one part of the sample underwent a completely static digital version of the test, referred to here as the Static Social Stress Test (Stat-DSST); the other part of the sample underwent a dynamic version of the same test, referred to as the Dynamic Social Stress Test (Dyn-DSST). Both task versions required participants to prepare and present five interview-style presentations. In particular, the sequence entailed, for each of the five speeches, three distinct phases: (i) the reading of the assignment; (ii) the preparation of the discourse within a maximum timeframe of 120 s; (iii) the presentation of the discourse within a time limit of 60 s. However, in the Stat-DSST version, the stimuli from the examining committee consisted of simple images of static people, devoid of sound and characterized solely by the emotional valence represented through facial expressions. In contrast, in the Dyn-DSST version, the committee was portrayed through realistic and simulated videos, which included—in addition to facial expressions similar to those in static images—non-verbal signs and a verbal request to the participants to provide the oral presentation.

Thus, through a multi-measurement methodological approach [41], this study tested the behavioral, autonomic, and neurophysiological (EEG) responses of healthy individuals performing interview-style presentations conducted in two different modalities, dynamically versus statically (Dyn-DSST and Stat-DSST).

Specifically, as behavioral data, we measured the response times (RTs) during the preparation phase (i.e., the time needed for a participant to prepare an interview presentation), which were considered as a cognitive metric for the indirect evaluation of the performance, the psychophysiological stress, and the level of activation of the participants’ nervous systems. As highlighted by earlier research, an increase in preparation time for a given performance may be considered an indicator of diminished performance and suboptimal stress regulation [42]. This observation aligns with the understanding that prolonged preparation periods may signify heightened cognitive loads and inadequate coping mechanisms, thereby suggesting a potential detriment to overall performance outcomes [43]. Based on the existing literature’s evidence [44], by examining stress regulation and resistance in the preparation phase, it is possible to unveil the anticipatory mechanisms that shape individuals’ abilities to effectively cope with stressors.

From the RTs recorded during the preparation phase, two behavioral indices reflecting stress regulation (Reg_Stress_) and stress resistance (Res_Stress_) were computed. As highlighted in the literature focused on high performance (e.g., in sports), it can be asserted that the preparation period leading up to a performance is crucial in evaluating stress management abilities. During this critical period, individuals engage in cognitive and emotional processes that significantly impact their subsequent performance [45].

Moreover, autonomic and EEG indices were considered to obtain a comprehensive picture of individuals’ stress management abilities in terms of the autonomic (arousal-related) and central (cortical-related) responses and provide multiple levels of explanation of the two experimental conditions [41].

Through this methodological approach, this study aims to address a significant gap in the current literature on the neurophysiological and behavioral regulation of stress in digital environments. Specifically, it examines the effects of dynamic (Dyn-DSST) and static (Stat-DSST) sensorimotor simulations on stress responses during simulated digital interviews. By employing a multimodal neuroscientific methodology that integrates EEG data and autonomic activity measurements, this research seeks to provide a comprehensive understanding of the underlying neurophysiological sensorimotor mechanisms. This approach elucidates the cognitive and emotional engagement elicited by dynamic versus static simulations. The primary objective is to advance beyond existing theoretical contributions by directly comparing these two distinct simulation modalities. In fact, unlike traditional studies, which often focus on static or less interactive settings, or those conducted in person, this research introduces an innovative experimental framework that mirrors the dynamic nature of modern digital environments.

## 2. Materials and Methods

### 2.1. Hypotheses

Starting from the study aim and methodological considerations reported above, firstly, we hypothesize that the SST implemented through the static modality could have—even considering the presence of limited external sensory input in time and space—a minor cognitive and emotional impact on the individual, and this, in turn, would translate into lower cognitive costs and greater stress regulation and resistance (Reg_Stress_ and Res_Stress_ scores) in the Stat-DSST compared to the Dyn-DSST group. Meanwhile, the dynamic condition—which requires participants to process not only verbal and/or written requests but also non-verbal and paraverbal components—could have a greater cognitive and emotional impact on participants, and this cost-demanding effect will translate into slightly lower behavioral performance in terms of the Reg_Stress_ and Res_Stress_ scores for the Dyn-DSST compared to the Stat-DSST group.

Secondly, for the behavioral data, we expect to find a positive relationship between the Reg_Stress_ and Res_Stress_ indices for both groups (Dyn-DSST and Stat-DSST). Indeed, as highlighted in the literature, effective stress management over extended periods and in the medium term, even in high-stakes contexts [46,47], can contribute to consolidating a “resilience reserve”, activated during acute and intense events, allowing individuals to maintain stability and proactively address challenges.

Thirdly, regarding EEG data, since the literature suggests an increase in delta band power in stressful conditions with a negative feeling [40], we suppose that a decrease in delta band power might reflect better stress management abilities and the development of such a “resilience reserve” in even more stress-inducing conditions, such as the Dyn-DSST compared to Stat-DSST. The same trend is expected for the low-frequency bands (theta and alpha band desynchronization), supporting the hypothesis that a decrease in these frequency bands may indicate higher required attentional control over the situation in the Dyn-DSST compared to the Stat-DSST group.

Fourthly, we hypothesize a significant increase in the gamma band frequency in the Dyn-DSST compared to the Stat-DSST group. Gamma band activity correlates with advanced cognitive processes and information processing from the surrounding context [48]; thus, an increase in gamma power in the Dyn-DSST compared to the Stat-DSST group may suggest increased cognitive involvement in dynamic compared to static interviews. Furthermore, this hypothesis finds additional support in the literature, which underscores how constant flows of audiovisual information stimulate the sensory and motor activation of the brain, triggering an increase in cognitive demand [49].

Finally, regarding autonomic data, we expect a significant increase in the EDA indices in the Dyn-DSST compared to the Stat-DSST group. This hypothesis is supported by existing evidence [28,29]; the simulated presence of the examination committee is likely to generate a more pronounced physiological response, deeply engaging the sensory stimulation and the emotional and social dimensions of judgment exposure.

### 2.2. Sample

A total of 52 healthy adults were selected as participants for this study (*M*_age_ = 25.25, SD_age_ = 3.435, age range: 22–35, N_male_ = 17, N_female_ = 35). They were recruited through a convenience sampling approach. The inclusion criteria were an age ranging from 22 to 35 years and normal or corrected visual and auditory acuity. Exclusion criteria were defined to prevent the participation of individuals showing clinically significant signs of distress and/or burnout. Further exclusion criteria included a history of neurological or psychiatric illnesses, current therapy with psychoactive substances potentially influencing decision-making or cognition, and major stressful life events in the last 6 months. Furthermore, the participants were checked for their clinical profiles: no psychopathological elements were observed for the depression construct (tested by Beck’s Depression Inventory II, total score equal to or less than 10 [50]).

The overall sample was randomly assigned to two subgroups balanced for age and gender. The first group, consisting of 26 individuals (*M*_age_ = 23.038, SD_age_ = 1.455, age range: 22–28, N_male_ = 10, N_female_ = 16), underwent the Dyn-DSST, while the second group (*M*_age_ = 27.462, SD_age_ = 3.432, age range: 22–35, N_male_ = 7, N_female_ = 19) underwent the Stat-DSST. To all participants, the Italian version of the Perceived Stress Scale 10 (PSS-10; [51]) was applied to exclude the presence of high stress levels during the thirty days preceding the tool’s administration (total score equal to or less than 27). No differences in the PSS were observed between the two groups.

No participant received any form of monetary or other compensation for their participation in the study. All participants took part in the study voluntarily and provided written informed consent. This research obtained approval from the Ethics Committee of the Department of Psychology, Catholic University of the Sacred Heart, Milan, Italy, and it was conducted in compliance with the Declaration of Helsinki (2013) and the GDPR—Reg. UE 2016/679 and its ethical guidelines.

### 2.3. Procedure

The participants were invited to a moderately illuminated room dedicated to the experiment, where they received the experiment’s instructions and were asked to sign the informed consent form.

The participants were fully informed that they would participate in a simulated and fictitious interview (and not a real job interview). They were told to imagine giving an interview during a selection test to access a stage or an ideal job position in front of an evaluation board.

Before the experiment, the participants were asked to sit and keep their feet firmly planted on the ground. Except for a computer positioned 100 cm away from the participant, on which the test was administered, all other personal technological devices (e.g., smartphones) were turned off to avoid interference with the EEG measurements. Following this preliminary introduction, the EEG and sensors for the detection of autonomic indices were applied to the participants.

After recording the baseline conditions with closed and open eyes, a web-based experiment management platform (Qualtrics XM platform; Qualtrics LLC., Provo, UT, USA) was used for the DSST’s administration. The task required the participants to prepare and present five different speeches, based on incrementally challenging requests.

The following instructions were given at the beginning of the task:

*“Imagine being in a selection test to access an internship or a job position that you care about. The representative of the organization informs you that there is only one available spot and 23 candidates interested in the position. You are scheduled for a selection interview where there will be an examining committee. You will need to prepare and orally present various speeches”.*

The participants were fully informed that, for each discourse, the preparation phase had a maximum time of 120 s, and the speech phase had a maximum time of 60 s. To achieve the highest score, the participants were informed that they were required to prepare their speech in the shortest time possible.

One part of the sample underwent the Dyn-DSST version, while the other half underwent the Stat-DSST version. The Dyn-DSST was conducted in a realistic context, characterized by environmental conditions and stimuli that mirrored the dynamic nature of the setting. In this specific condition, the evaluation committee consisted of real individuals who were video-recorded, contributing to the authentic transmission of visual, auditory, motor, verbal, and non-verbal stimuli. This methodology favored a more ecologically valid approach. Conversely, the Stat-DSST version involved an evaluation committee composed of static images through a designed reproduction, aimed at creating a sense of distance and separation from the participants. This methodological choice was implemented to explore the participants’ responses to less immediate and more distant stimuli. In this context, static images played a key role in creating an assessment environment that differed from the immediate reality, allowing an analysis of the participants’ cognitive abilities and emotional responses not in the face of real stimuli but in response to remote stimuli.

Furthermore, during the task, the reactions of the evaluation committee incorporating various emotional states were incrementally manipulated. The increase in complexity and the growing conditions of stress were deliberately induced through a dual approach: (i) the complexity of the content of the requests during the preparation phase was heightened (e.g., D1, optimal self-presentation; D5, decision-making in disagreement with the group); (ii) the negative intensity of the non-verbal communication from the evaluation committee to which each participant was exposed was escalated (e.g., variations in speech speed, emphasized pauses, greater expressive articulation, a rigid posture, and the more pronounced use of gestures and facial expressions). External judges carefully examined and evaluated the stimuli to ensure that they were consistent and reliable according to the intended objectives and outcomes of the experiment.

**Figure 1:**
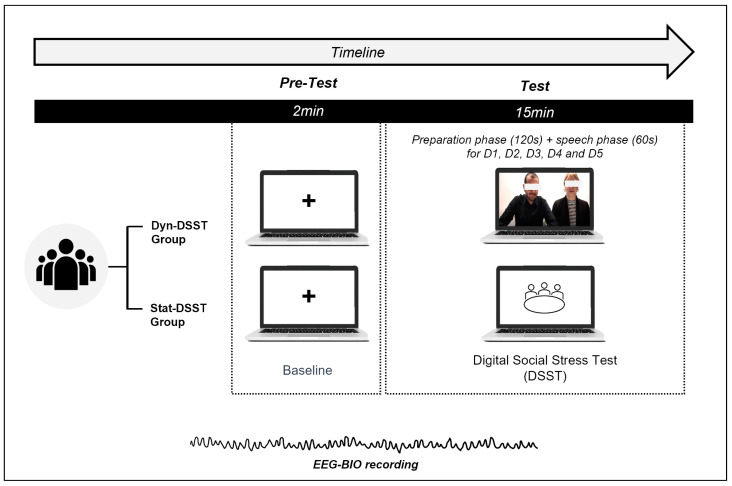
Graphical description of the experimental procedure. EEG and autonomic activity were monitored from the baseline throughout the task together with behavioral data recording.

Table 1 shows the content of the speech requests and the corresponding evaluation board’s emotional states for each discourse (Table 1). Figure 1 depicts the experimental procedure, which lasted approximately 45 min (Figure 1).

### 2.4. Behavioral Data Processing

The performance of each participant was automatically recorded by the system, collecting RTs for the most critical phase of the interview (preparation phase) of every discourse, as an indirect measure of their performance. These data were subsequently processed offline to obtain the stress regulation and acute stress resistance scores (respectively, Reg_Stress_ score and Res_Stress_ score).

The experimenter employed both objective and qualitative criteria to assess the relevance, coherence, and appropriateness of each discourse. Any discursive production in which the content was deemed non-equivalent in terms of productivity—characterized by substance-lacking content, the inclusion of personal arguments unrelated to the task, and inconsistency with the requirements of each discourse—was excluded from the analysis. Through this process, the contents of the five analyzed discourses were deemed equivalent, allowing for an objectively comparable measure of performance derived from the preparation phase.

The scoring assignment (Table 2) was established through a thorough analysis of the normal distribution of the results obtained during a preliminary pilot test (N = 131). This pilot test was conducted on a representative sample, comprising participants selected based on specific inclusion criteria analogous to those of the present study.

Specifically, for Reg_Stress_ score, a score from 1 to 5 was assigned based on each participant’s performance in the preparation phase for each discourse based on the normal distribution reported in the table. A higher score indicated a better ability to regulate stress. For Res_Stress_ score, a score from 1 to 5 was assigned based on the absolute value difference between the averages of the preparation times for D4–D5, compared to the average, also expressed as an absolute value, and the overall preparation performance for the five discourses. A higher score indicated greater stress resistance. Subsequently, both scores, Reg_Stress_ score and Res_Stress_ score, were transformed into deciles.

### 2.5. EEG Data Acquisition and Processing

After setting up the environment, the participants underwent an EEG assessment of the resting state. EEG data were collected with eyes both open and closed (120 s for each condition).

The EEG data related to the baseline and recordings during the test, further divided into the five different speeches and the preparation and execution phases, were acquired using an 18-channel DC amplifier (SYNAMPS system) with the support of the NEUROSCAN 4.2 acquisition software. An ElectroCap with Ag/AgCl electrodes was used for EEG recording, placed on 18 active sites of the scalp following the 10/20 electrode placement system [52], with reference to the earlobes.

In addition, two electrooculographic electrodes (EOG) were placed above and below the left eye of each participant to remain outside the visual field. Data were recorded at a sampling frequency of 1000 Hz and with a 50 Hz notch input filter. Before data collection, the electrode impedance was monitored for each subject, ensuring that it remained consistently below 5 kΩ.

Subsequently, data related to the eyes-open and eyes-closed resting states and related to the preparation phase of every discourse were processed offline (IIR bandpass filter 0.1–50 Hz, 48 db/octave) using the Vision Analyzer version 2.0 software (Brain Products GmbH, Gilching, Germany) and corrected by an ICA-based algorithm [53]. After EOG correction and careful visual inspection, only segments free of muscle artifacts, eye artifacts, or interfering disturbances were considered. The data were epoched using a time window of 2000 ms. This approach ensured the integrity and quality of the collected EEG data. Artifact-free data were then used to calculate condition-specific power density spectra (PSD) using a Fast Fourier Transform (Hamming window, resolution: 0.5 Hz). The average PD for each band was extracted for each considered phase as follows: delta (0.5–3.5 Hz); theta (4–7.5 Hz); alpha (8–12.5 Hz); beta (13–30 Hz); and gamma (30.5–50 Hz).

For the statistical analysis, four regions of interest (ROIs) were derived from eight electrodes—frontal 1 (F1: F3; F7), frontal 2 (F2: F4; F8), temporo-parietal 1 (TP1: T7; P3), and temporo-parietal 2 (TP2: T8; P4)—covering the left and right frontal and temporoparietal neuroanatomical regions. Additionally, all task-related data were normalized to the eyes-open baseline of each participant using the following formula:

TR_PSD_ = ((PSD_task_ − PSD_open-bl_)/PSD_open-bl_)

### 2.6. Autonomic Data Acquisition and Processing

To continuously monitor the autonomic activity of each participant, the Biopac MP 150 system from Biopac Systems Inc. (USA) was employed. For ECG recording, two electrodes were placed on the lower wrist, with the positive pole on the left arm and the negative pole on the right arm. The ECG signal was sampled at 1000 Hz using the Biopac Acknowledge 3.7.1 software, following the manufacturer’s instructions. To eliminate any motor and ocular artifacts, the signal underwent low-pass filtering at 35 Hz. Subsequently, the signal was converted into the HR, expressed in beats per minute (bpm), using predefined values for human rest. From these data, inter-beat interval values were derived and then converted into milliseconds, to calculate the heart rate variability (HRV).

Regarding the measurement of the electrodermal activity, two electrodes were placed on the palm of each participant’s non-dominant hand, at the thenar and hypothenar reference points. The EDA signal was sampled at 1000 Hz and subjected to a low-pass filter at 10 Hz for artifact removal, to obtain the skin conductance level (SCL) values and skin conductance responses (SCR).

### 2.7. Data Analysis

For the behavioral data, two one-way ANOVAs were applied with the *group* (2: Dyn-DSST, Stat-DSST) as the independent variable and the Reg_Stress_ and Res_Stress_ scores as the dependent variables. Moreover, correlation analyses (Pearson correlation coefficients) were applied to explore the relation between the Reg_Stress_ and Res_Stress_ scores.

Subsequently, for the EEG results, five mixed repeated-measures ANOVAs considering the *group* (2: Dyn-DSST, Stat-DSST) as the between-subject independent variable and the *ROI* (4: F1, F2, TP1, TP2) and *discourse* (5) as the within-subject independent variables were carried out for the five different frequency bands (delta, theta, alpha, beta, and gamma).

Finally, for the autonomic data, four mixed repeated-measures ANOVAs with the *group* (2: Dyn-DSST, Stat-DSST) as a between-subject independent variable and *discourse* as a within-subject independent variable were applied to the following autonomic indices considered as dependent variables: SCR, SCL, HR, and HRV.

For all ANOVA tests, the degrees of freedom were corrected by the Greenhouse–Geisser epsilon when appropriate. Simple effects for significant interactions were further checked via pairwise comparisons, and Bonferroni correction was used to reduce multiple comparisons’ potential biases. The size of the statistically significant effects was estimated by computing the eta-squared (*η*^2^) indices. The threshold for statistical significance was set at α = 0.05. For the statistical analysis, IBM SPSS 29 (IBM Corp., Chicago, IL, USA) was used. In a preliminary analysis, we tested for potential differences related to gender and no statistically significant main or interaction effects were found; thus, this variable was not included in the below-reported analyses.

## 3. Results

### 3.1. Behavioral Results

**Figure 2:**
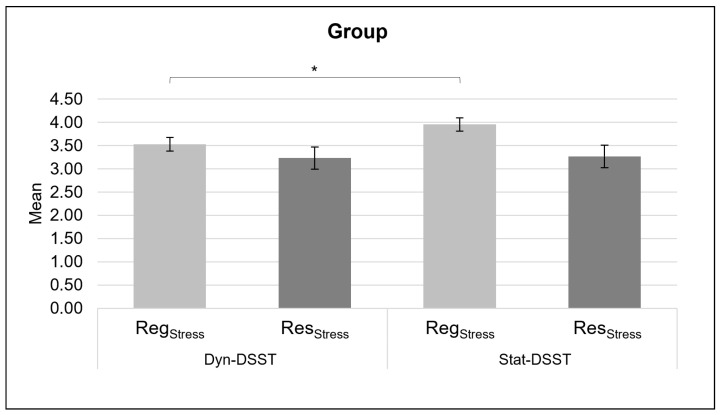
*Behavioral results*. The bar graph shows statistically significant differences in the behavioral stress scores (Reg_Stress_ score and Res_Stress_ score) for each group (Dyn-DSST and Stat-DSST). Bars represent ± 1 standard error and stars (*) mark statistically significant comparisons.

Considering the analysis performed on the Reg_Stress_ score, a significant main effect was found for the group (*F*(1,50) = 4.188, *p* = 0.046, *η*^2^ = 0.077), with a higher mean for the Stat-DSST group compared to the Dyn-DSST group (Figure 2). No significant effects were found for the Res_Stress_ score.

#### Correlational Results between Behavioral Scores

**Figure 3:**
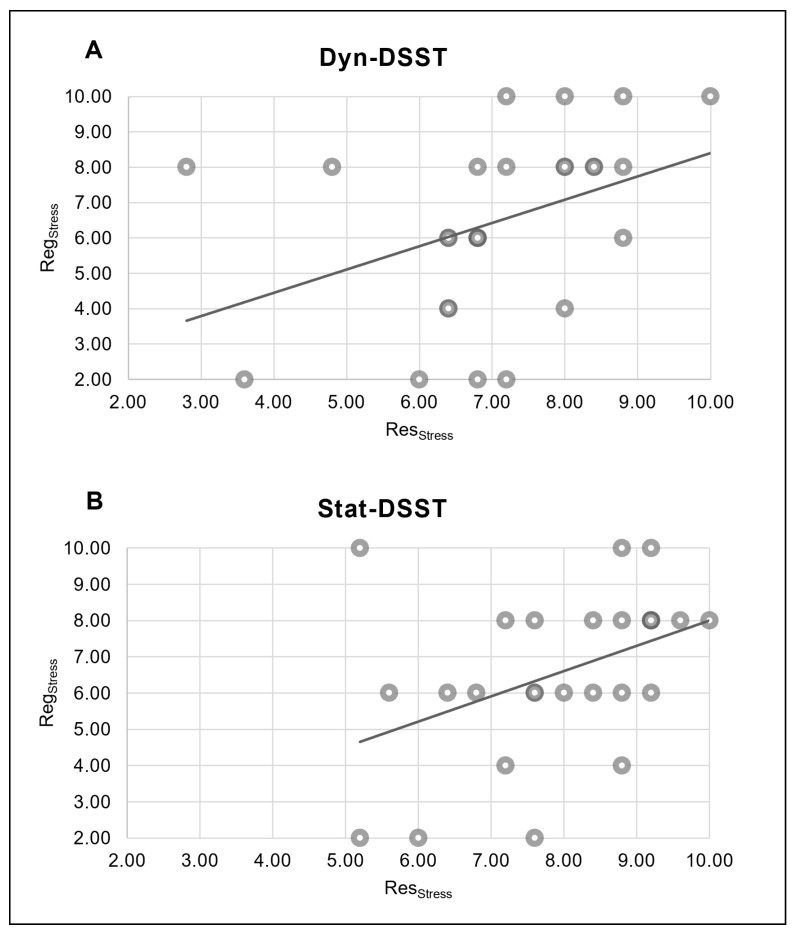
*Pearson correlations between behavioral scores*. (**A**) The scatter plots display a significant positive correlation between the Reg_Stress_ score and Res_Stress_ score in the Dyn-DSST group. (**B**) The scatter plots display a significant positive correlation between the Reg_Stress_ score and Res_Stress_ score in the Stat-DSST group.

The Pearson correlation analyses also revealed a positive correlation between the Reg_Stress_ and Res_Stress_ scores for the Dyn-DSST group (r = 0.401, *p* = 0.042) (Figure 3A). Similarly, in the Stat-DSST group, a positive correlation was observed between the Reg_Stress_ and Res_Stress_ scores (r = 0.418, *p* = 0.033) (Figure 3B).

### 3.2. EEG Results

The following sections show the results of the repeated-measures ANOVAs performed with the *ROI* (4: F1, F2, TP1, TP2) and *discourse* (5) as the independent within-subject factors and the *group* (2: Dyn-DSST, Stat-DSST) as a between-subject factor, for the different frequency bands (delta, theta, alpha, beta, and gamma).

#### 3.2.1. Delta

**Figure 4:**
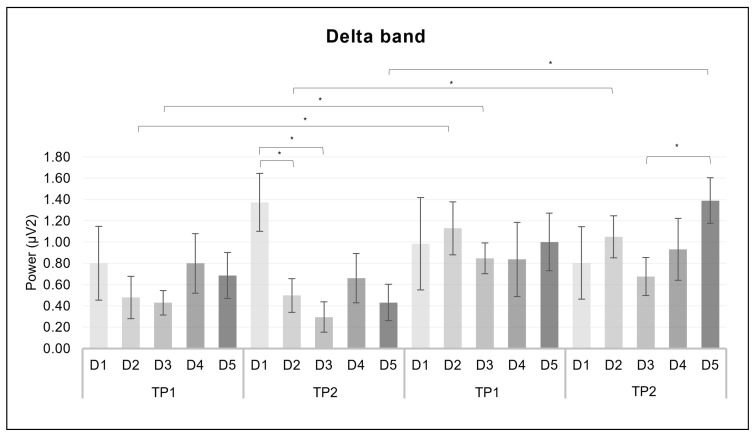
*EEG delta results*. The bar graph shows significant differences for the delta band in *group* × *ROI* × *discourse*. Bars represent ± 1 standard error and stars (*) mark statistically significant comparisons.

For the delta band, a significant interaction effect was revealed for *group* × *ROI* × *discourse* (*F*_(4.2, 144)_ = 2.606, *p* = 0.035, *η*^2^ = 0.071) (Figure 4).

Considering the comparison between the two groups, the significant pairwise comparisons revealed lower mean values for the Dyn-DSST group compared to the Stat-DSST group in TP1 (*p* = 0.049) as well as in TP2 (*p* = 0.036) during D2. Moreover, the Dyn-DSST group exhibited lower mean values than the Stat-DSST group in TP1 during D3 (*p* = 0.030) and in TP2 during D5 (*p* = 0.001). For the Dyn-DSST group, the pairwise comparisons revealed that D1 showed higher mean values than D2 (*p* = 0.028) and D3 (*p* = 0.007) in TP2. Finally, for the Stat-DSST group, lower mean values were observed for D3 than D5 in TP2 (*p* = 0.002).

#### 3.2.2. Theta

**Figure 5:**
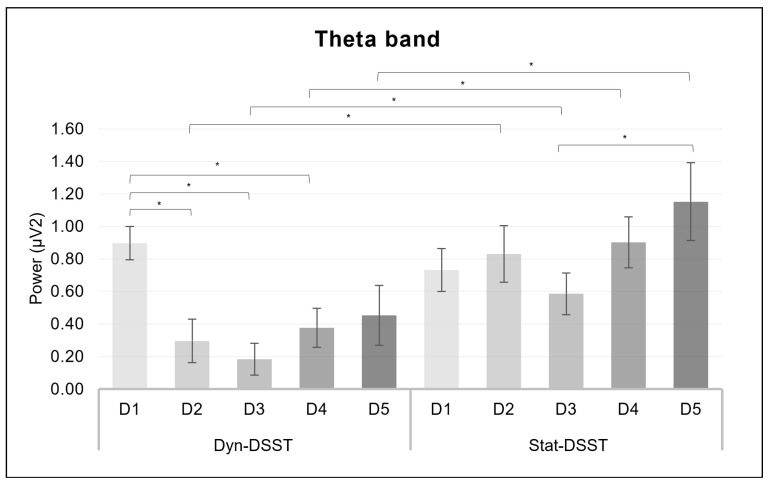
*EEG theta results*. The bar graph shows significant differences for the theta band in *group* × *ROI* × *discourse*. Bars represent ± 1 standard error and stars (*) mark statistically significant comparisons.

For the theta band, a significant interaction effect was revealed for *group* × *ROI* × *discourse* (*F*_(6, 199_._4)_ = 3.402, *p* = 0.003, *η*^2^ = 0.093) (Figure 5). Firstly, the pairwise comparisons showed that the mean values for the Dyn-DSST group were significantly lower than those for the Stat-DSST group during D2 (*p* = 0.020), as well as during D3 (*p* = 0.018), D4 (*p* = 0.012), and D5 (*p* = 0.027), in TP2. For the Dyn-DSST group, higher mean values were observed for D1 compared to D2 (*p* = 0.001), D3 (*p* ≤ 0.001), and D4 (*p* = 0.019) in TP2. Furthermore, for the Stat-DSST group, the pairwise comparisons showed significantly higher activity for D5 than D3 (*p* = 0.044) in F2.

#### 3.2.3. Alpha

**Figure 6:**
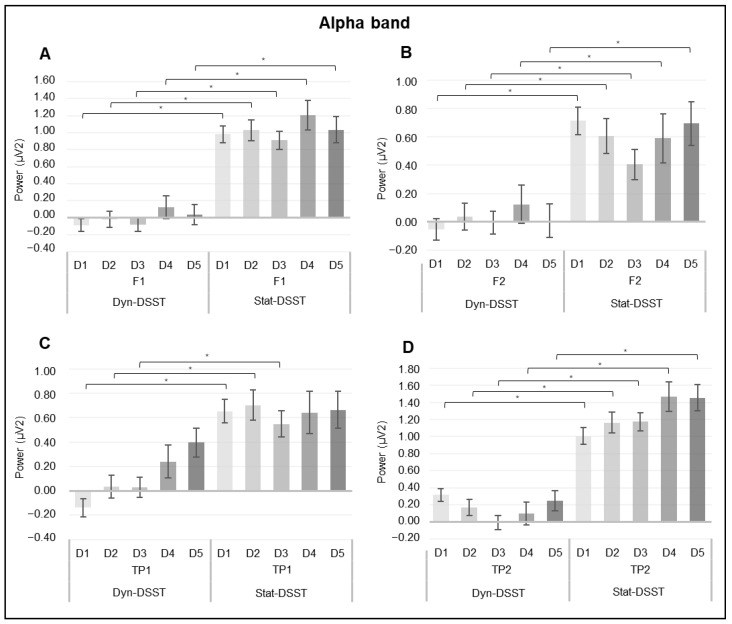
(**A**–**D**) *EEG alpha results.* The bar graph shows significant differences for the alpha band in *group* × *ROI* × *discourse*. (**A**) The bar graph displays the significant decrease in the alpha values in the Dyn-DSST compared to the Stat-DSST group for F1 during all discourses. (**B**) The bar graph displays the significant decrease in the alpha values in the Dyn-DSST compared to the Stat-DSST group for F2 during all discourses. (**C**) The bar graph displays the significant decrease in the alpha values in the Dyn-DSST compared to the Stat-DSST group for TP1 during D1, D2, and D3. (**D**) The bar graph displays the significant decrease in the alpha values in the Dyn-DSST compared to the Stat-DSST group for TP2 during all discourses. For all graphs, bars represent ± 1 standard error, and stars (*) mark statistically significant comparisons.

For the alpha band, a significant interaction effect was also revealed for *group* × *ROI* × *discourse* (*F*_(4_._164, 137_._4)_ = 2.479, *p = 0.045*, *η*^2^ = 0.070) (Figure 6).

Firstly, considering the comparison between the two groups concerning F1, the pairwise comparisons showed that the Dyn-DSST group exhibited decreased alpha activity compared to the Stat-DSST group in F1 during all of the discourses: D1 (*p* ≤ 0.001), D2 (*p* ≤ 0.001), D3 (*p* ≤ 0.001), D4 (*p* ≤ 0.001), and D5 (*p* ≤ 0.001) (Figure 6A). Similar results were observed in the comparison between the two groups with reference to F2. The Dyn-DSST group exhibited decreased alpha activity compared to the Stat-DSST group in F1 during all of the discourses: D1 (*p* ≤ 0.001), D2 (*p* = 0.005), D3 (*p* = 0.009), D4 (*p* = 0.018), and D5 (*p* ≤ 0.001) (Figure 6B).

Considering TP1, the Dyn-DSST group showed decreased alpha activity compared to the Stat-DSST group during D1 (*p* ≤ 0.001), D2 (*p* = 0.001), and D3 (*p* = 0.005) (Figure 6C). Lastly, similarly to the first two sets of results, concerning TP2, the Dyn-DSST group exhibited a decrease in alpha activity compared to the Stat-DSST group during all discourses: D1 (*p* = 0.001), D2 (*p* ≤ 0.001), D3 (*p* ≤ 0.001), D4 (*p* ≤ 0.001), and D5 (*p* = 0.003) (Figure 6D).

#### 3.2.4. Beta

**Figure 7:**
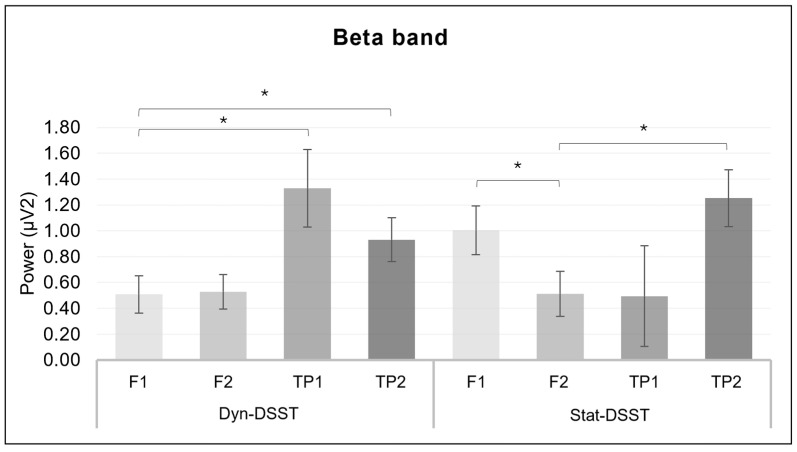
*EEG beta results.* The bar graph shows significant differences for the beta band in group × ROI. Bars represent ± 1 standard error and stars (*) mark statistically significant comparisons.

For the beta band, a significant interaction effect was revealed for *group* × *ROI* (*F*_(1.7, 56)_ = 5.275, *p* = 0.011, *η*^2^ = 0.138) (Figure 7).

For the Dyn-DSST group, the pairwise comparisons revealed significantly lower mean values for F1 compared to TP1 (*p* = 0.025) and TP2 (*p* = 0.048). For the Stat-DSST group, the mean values for F1 were higher than for F2 (*p* = 0.016). Furthermore, within the Stat-DSST group, the pairwise comparisons showed significantly higher activity in F2 than in TP2 (*p* = 0.006).

#### 3.2.5. Gamma

**Figure 8:**
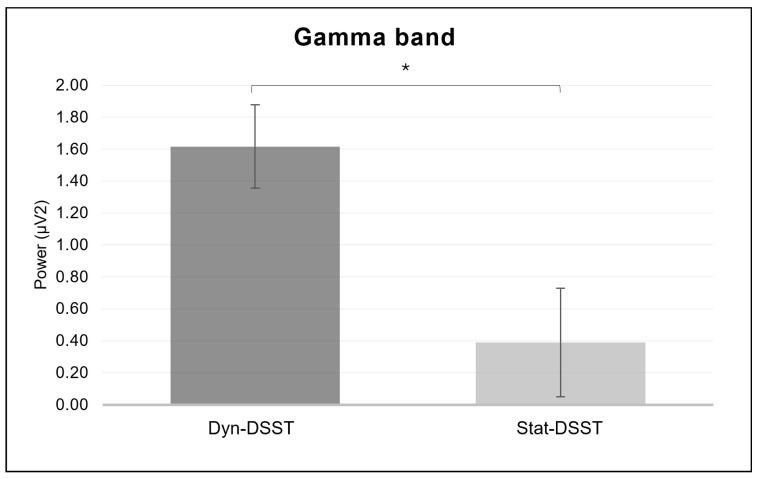
*EEG gamma results.* The bar graph shows significant differences for the gamma band in the group. Bars represent ± 1 standard error and stars (*) mark statistically significant comparisons.

For the gamma band, a significant main effect was revealed for the *group* (*F*_(1, 33)_ = 8.258, *p* = 0.007, *η*^2^ = 0.200), with higher mean values for the Dyn-DSST group compared to the Stat-DSST group (Figure 8).

### 3.3. Autonomic Results

**Figure 9:**
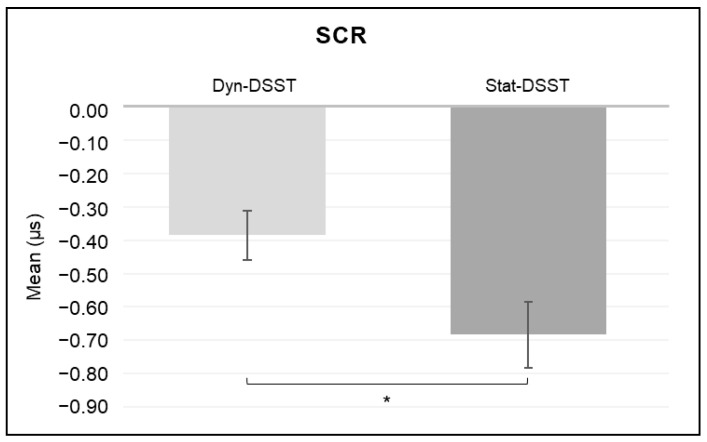
*Autonomic results.* The bar graph shows significant differences in the SCR for the group. Bars represent ± 1 standard error and stars (*) mark statistically significant comparisons.

For the SCR, a significant main effect was revealed for the *group* (*F*_(1, 31)_ = 5.813, *p* = 0.022, *η*^2^ = 0.158) (Figure 9), with higher mean values for the Dyn-DSST group compared to the Stat-DSST group.

No other significant results were found.

## 4. Discussion

This study explored the behavioral, autonomic, and neurophysiological (EEG) responses of healthy individuals performing interview-style presentations conducted in two different modalities: a dynamic versus a static version (Dyn-DSST and Stat-DSST). During the interview preparation phase, RTs were collected and two behavioral indices expressing individuals’ stress regulation and resistance abilities (Reg_Stress_ and Res_Stress_) were computed. Moreover, EEG and autonomic indices were simultaneously recorded throughout the task, using a multi-measurement methodological approach [41]. Such an approach was exploited to provide multiple levels of explanation for the two experimental groups and to obtain a comprehensive picture of individuals’ stress management abilities in terms of the autonomic (arousal-related) and central (cortical-related) responses.

The behavioral results suggested that the participants undergoing the static version (Stat-DSST group) exhibited higher behavioral performance in terms of Reg_Stress_ compared to the Dyn-DSST group, perhaps because the static condition was perceived as less stressful. However, in both groups, the more the individuals were able to regulate their stress, the more they could resist it even in conditions of acute stress.

Similarly, as hypothesized, the EEG findings displayed a distinct contribution of the low- and high-frequency bands for the two experimental groups, with a decrease in the delta, theta, and alpha band power in the Dyn-DSST compared to the Stat-DSST group. This result mirrored the need for greater cognitive regulation control effort in the Dyn-DSST group. On the other hand, the significant increase in the gamma band power in the Dyn-DSST compared to the Stat-DSST group was interpreted as lower self-awareness in the static condition.

Finally, an increase in the SCR was observed in the Dyn-DSST compared to the Stat-DSST group, suggesting increased emotional engagement in the dynamic environment compared to the more static digital interaction.

These findings will be discussed below considering the existing literature.

First, it was observed that there was a significant and positive correlation between the Reg_Stress_ and Res_Stress_ indices regardless of the experimental group (both in Dyn-DSST and Stat-DSST). Thus, individuals capable of effectively regulating their stress demonstrated greater resistance during the two stressful interview modalities, irrespective of the mode of interview administration.

Participants undergoing the static version (Stat-DSST group) exhibited higher behavioral performance in terms of Reg_Stress_ compared to the Dyn-DSST group. This evidence could be interpreted from the perspective of the perceived stress levels, suggesting that, in the static condition, there was a lower perception of stress compared to the dynamic condition. In particular, the static image of the committee could have limited the implicit motor activation associated with simulating interpersonal dynamics, resulting in a greater sense of control and predictability, ultimately leading to better overall stress response regulation and the reduced perception of issues derived from dynamic variables [54]. Conversely, in the Dyn-DSST group, the exposure to real-life simulation videos could heighten the stress response as the participants need to adapt to new information, non-verbal cues, and real-life movements, thus reducing the overall predictability of the stimuli.

Another potential interpretation of this effect could be the decreased awareness of stress-inducing stimuli in the static mode. This explanation is in line with former works suggesting that fully remote conditions could weaken empathy levels [55] and work engagement [56] and ultimately affect social interactions at an emotional level [57]. It is reasonable to suggest that the reduced sensation of “physical presence” caused by the static digital setting contributes to the decreased awareness of the stress levels. This perspective could be attributed to the perception of the other, which, due to the physical distance and mediated nature of digital communication, may induce a different representation of the context. Indeed, the perception of distance could contribute to a less threatening view of the evaluative context, thus influencing the overall perception of the stressful stimuli. In this way, the digital static medium could play a significant role in modulating psychological reactions, consequently impacting the stress regulation response.

Instead, for Res_Stress_, no significant differences between the groups were observed. A possible reason for this lack of effect could be that behavioral measures do not always reflect healthy samples’ performance [58] or the subthreshold stress management mechanisms with the same sensitivity as neuroscientific measures. Thus, the results obtained from the multimethod neuroscientific approach offer added value by not only highlighting but also illustrating and unraveling the implicit stress resistance of the individuals in the Dyn-DSST and Stat-DSST groups, which behavioral data alone may fail to capture.

This outcome suggests a strict link between the ability to manage stress and the ability to endure it during stressful conditions: the more individuals can regulate stress, the more they can resist it, even in conditions of acute stress. Indeed, according to Lazarus and Folkman’s stress management theory [59,60], stress regulation may act as a mediator, influencing the perception of a situation and, consequently, stress resistance. We demonstrate that this relation is confirmed both in completely digitized static contexts and in digital dynamic interaction situations.

Considering the EEG results, first, a significant reduction in delta band power was found in the Dyn-DSST compared to the Stat-DSST group. This decrease was observed, in particular, in the temporo-parietal area and intensified with the increased complexity of the task, which was consistent with a rise in negative emotional valence associated with the examining committee’s verbal and non-verbal expressions.

Potential explanations for this phenomenon may be found in the connection between diminished delta power in the temporo-parietal area and an enhanced ability to regulate stress [40]. Reduced delta band activation in the temporo-parietal region may indicate a greater need for regulatory effort, efficiency in cognitive processes, and better emotional control during stressful situations in the dynamic administration condition. This result could be considered an indicator of neurophysiological adaptation, suggesting that the realistic interview administration mode may lead to increased cognitive effort in participants, especially in the face of complex requests and negative emotional charges. These results could also be considered based on the recent literature, which highlights how digital simulations require continuous information processing and responses to external inputs in real time [49], involving the sensorimotor systems and an increasing cognitive load and brain activation in the lower temporo-parietal area [61,62].

Such an interpretation is consistent with the second set of significant results from our study. The results indicating a decrease in delta in the temporo-parietal regions that is more pronounced in the Dyn-DSST group at D2, D3, and D5 are consistent with the theta results. Indeed, similar patterns were observed for the theta band, with a decrease in the right temporo-parietal region’s power in the Dyn-DSST compared to the Stat-DSST group in discourses D2, D3, D4, and D5. Furthermore, an additional set of significant results highlights that, within the Dyn-DSST group, a decrease in this band was observed in D2, D3, and D4 compared to D1. Overall, discourses with a greater focus on decision-making demands, which were more stressful and implied a negative evaluation by the committee (neutrality, boredom, impatience, aversion), may necessitate, especially in the dynamic exposure condition, additional regulatory effort and, accordingly, the greater employment of stress management resources.

Thirdly, mirroring the low-frequency patterns in the Dyn-DSST group, a significant increase in delta band power during a discourse characterized by higher demands and emotional complexity (D5) compared to a lower processing context (D3) was found for the Stat-DSST group in the right temporo-parietal region. This trend was similarly identified in the theta band.

These findings not only reinforce the former findings observed for the Dyn-DSST, for which higher cognitive effort is needed, but also suggest that the low-frequency variations observed in the right temporo-parietal region regulate the adaptive response to stress differently based on whether the person is exposed to a dynamic or static condition. An interpretation of this phenomenon could be found in the context of remote communication, which might influence the subjective perception of emotional demands. Indeed, in a fully static and remote environment, the perceived demands may undergo differentiated filtration, attenuating the perception of the challenge associated with progressively complex requests. Future studies could deepen this evidence and test whether the physical distance may act as a mitigating factor, rendering the cognitive and emotional demands more manageable, even in the presence of an objectively more stressful condition.

Fourthly, the lower power of the alpha band in both the left and right frontal and temporo-parietal regions for the Dyn-DSST compared to the Stat-DSST group confirms the previously discussed results, according to which the dynamic condition requires greater cognitive effort and attentional regulation, compared to the static condition. In fact, due to the phenomenon of cortical idling, the lower presence of alpha waves corresponds to an increase in an individual’s attentional engagement [63,64]. On the other hand, we also detected an increase in the beta band over specific ROIs for both experimental groups, perhaps suggesting the involvement of conscious attentional processes for both conditions. Since beta varies mainly in relation to attentional processes [33] and less directly in relation to stress regulation, the condition and type of speech do not seem to influence beta activation.

Finally, a significant increase in the gamma band power in the Dyn-DSST compared to the Stat-DSST group was found. These data align with theories associating the gamma band with advanced cognitive processes and the processing of information from the surrounding context. This finding could also be interpreted considering sensorimotor simulation, for which the increase in gamma power in the Dyn-DSST group may indicate higher cognitive involvement, engagement, and interaction during dynamic simulations compared to job interviews conducted entirely remotely. Plausible interpretations of this result can be derived from the literature’s evidence [65], suggesting a relationship between lower levels of gamma activity in the Stat-DSST group and reduced self-awareness. This connection could imply that the absence of a physical, real, or simulated presence might unfavorably influence individuals’ awareness regarding their actions, the detection of demands, and the surrounding context.

Further support for the interpretation of the EEG findings, and particularly for the gamma band power, can be derived from the autonomic results, where an increase in the SCR is observed in the Dyn-DSST compared to the Stat-DSST group. This increase could be interpreted as a signal of heightened emotional responsivity [28,29] in the dynamic environment compared to the more distant digital interaction, suggesting more pronounced emotional involvement in the former condition. In line with the literature, this result highlights the potential significance of the reduced sensorimotor simulation induced by static conditions on peripheral activation, representing involvement that is not only cognitive but also emotional [66,67].

To summarize, the comparison of these stressful interview conditions allowed us to identify their distinctive features: in the static condition, the participants displayed less cognitive effort and greater overall economy of resources and stress management, but there was also less awareness of the situation and less engagement by the “remote” condition; in the dynamic condition, the participants showed a greater sense of awareness and presence, along with emotional engagement related to the context of real perceived stress.

*Which of these two conditions is more ideal for interviews in real-world settings?* The answer relies on several potential expected outcomes, such as the degree of participation that we hope to observe in the person being interviewed and the cognitive and emotional “costs” that the person will have to bear to be essentially “present”.

Rather than being related to the degree of stress derived from the interview modality, the differences observed in this study are mediated (as a mediator) by the different “perceptions” (and therefore by the perceived stress levels) induced by the two conditions.

Furthermore, the findings obtained in this study allow us to conclude that sensorimotor integration is closely related to both stress regulation and stress resilience mechanisms. Prior research has shown that effective sensorimotor integration enables individuals to rapidly process sensory information and execute appropriate motor responses, which can help to mitigate the impact of stressors [18,24].

The behavioral data from our study showed that the participants in the Stat-DSST group, who faced less demanding sensorimotor integration tasks, had better stress regulation, as indicated by their reduced RTs and higher regulation scores. Stress resilience, as an integral part of the stress response, is also influenced by sensorimotor integration. When individuals are exposed to dynamic and unpredictable environments, as simulated in the Dyn-DSST, their ability to integrate sensory inputs and coordinate motor responses becomes crucial [17].

The increased cognitive load and emotional involvement observed in the Dyn-DSST group reflect the greater demands placed on their sensorimotor integration processes. This higher demand can deplete the cognitive resources more quickly, potentially compromising individuals’ stress resilience. In contrast, in the Stat-DSST group, the reduced sensory input allowed for more efficient sensorimotor integration, preserving the participants’ cognitive resources and improving their stress resilience.

In conclusion, this study provides preliminary evidence of a potential association between sensorimotor integration and both stress regulation and resilience. This association is indicated by the varied effects observed in both the dynamic and static digital interview conditions. Efficient sensorimotor integration may contribute to the ability to respond to stressors in a timely and appropriate manner, which in turn could potentially support both immediate stress management and the development of long-term stress resilience.

### Limitations

To the best of our knowledge, this study constitutes the first effort to compare the multilevel effects of dynamic versus static interview modalities (with a modified version of the TSST); however, some limitations should be taken into consideration.

First, future studies must integrate both the preparation and speech phases, providing a comparison between the anticipatory and the actual stress regulation and resistance performance in a complete and integrated manner.

Secondly, the groups were not balanced in terms of the hormonal states of the female participants. In fact, in this study, we performed preliminary verbal verification to ascertain the presence of any potential hormonal alterations that could have influenced the participants’ performance. This initial step was integral in attempting to control for biological variables that might confound the results. However, this approach has its limitations.

The reliance on verbal verification alone is insufficient to accurately assess hormonal fluctuations and their impacts. Therefore, a significant limitation of our study is the lack of comprehensive hormonal monitoring. This gap underscores the need for future research to incorporate detailed and systematic assessments of hormonal levels. Such evaluations should employ more rigorous methodologies, including biochemical analyses, to provide a clearer understanding of how hormonal fluctuations might affect participants’ neurophysiological and psychological responses during stress-inducing tasks.

Thirdly, the analysis of the EEG cortical response was derived from limited ROIs, and a more detailed examination of this phenomenon is required, including a thorough investigation of the cortical localization of brain oscillations using, for example, neuroimaging methods like functional near-infrared spectroscopy, which has been shown in multiple prior studies to be an accurate means of data collection during interactive dynamics [68].

Creating a condition of real interaction with a two-way interactive exchange between inter-agents could be of further interest, using the hyperscanning paradigm, as presented in our previous studies, focusing on the neurophysiological synchronization between the individual and the members of the examining committee (thus, with a multi-subject hyperscanning paradigm [69]).

Finally, the role of high-frequency bands, particularly the gamma band, should be better explored in future studies, given that the current results suggest a possible reduction in self-awareness in the static condition compared to the dynamic one. In terms of practical applications, further in-depth analysis would prove useful and interesting since it would enable us to provide recommendations to institutions that regularly conduct interviews (e.g., universities or business organizations) regarding which settings are the most stimulating and which place candidates in the best position.

## 5. Conclusions

In conclusion, the present study investigated how sensorimotor simulation in job assessments represents a significant innovation in the field of personnel evaluation. It has delineated the behavioral, autonomic, and neurophysiological impacts of two distinct interview modalities: dynamic versus static (Dyn-DSST and Stat-DSST). Through a multimethod neuroscientific approach, individuals’ stress regulation and resilience during these demanding circumstances were explored, examining how the interview format may influence their capacity to manage stressors. Furthermore, understanding how specific features of the virtual environment affect individuals’ sensorimotor experiences and neurophysiological responses is crucial in devising effective stress management strategies during online evaluations and enhancing the validity and reliability of such tools in personnel selection.

The examination of stress responses in different interview settings holds implications for the refinement of interview methodologies and the enhancement of the overall experience for both interviewers and interviewees. The findings and subsequent discussion can enrich the broader discourse on stress psychology, offering nuanced insights into stress resilience within the specific realm of job interviews.

### Proposals and Recommendations

To conclude, this article proposes several recommendations to advance the theoretical and practical understanding of stress management in digital environments.

Firstly, we suggest that future research should continue to utilize and refine multimodal neuroscientific methodologies, such as integrating EEG data with autonomic measures, to gain a more comprehensive understanding of the neurophysiological bases of stress responses. 

Secondly, we recommend that subsequent studies expand their scope to include a wider range of digital contexts and simulation modalities. This will allow for a better understanding of how different degrees and types of dynamic interaction influence stress and the cognitive load.

Thirdly, we propose that organizations and institutions involved in personnel selection and training should adopt dynamic simulation tools that closely mirror real-world scenarios. This will enhance the ecological validity of assessments and training programs, thereby improving the accuracy of assessments and the effectiveness of stress management interventions.

Fourthly, we suggest that autonomic index collection should be integrated into these simulations to provide real-time data on stress levels, enabling more immediate and personalized stress regulation strategies.

Lastly, we strongly recommend the development of training programs aimed at enhancing individuals’ cognitive and emotional regulation abilities in dynamic environments. These programs should incorporate evidence-based techniques to equip individuals with the skills necessary to effectively manage stress in high-pressure situations.

In conclusion, these practical recommendations highlight the importance of ongoing methodological innovations, broader contextual exploration, and the practical application of research findings to enhance stress management practices in digital environments.

## Figures and Tables

**Table 1:** Description of the speech requests and emotional states of the examining committee for each discourse.

Discourse	Speech Request	Evaluation Committee Emotional Display
1	“We ask you to prepare the best presentation of yourself.”	Friendly
2	“We ask you to describe a critical situation in which you encountered difficulties in making a decision on your academic/professional career or during previous internships.”	Neutral
3	“We ask you to describe a critical situation in the academic/professional context where you faced a hard challenge and you needed to take a decision completely alone, without having the support of anyone in making a decision.”	Bored
4	“We ask you to describe a situation in which you found yourself taking a critical decision alone in the academic/professional context, by taking full responsibility of it and in complete disagreement with the rest of the group.”	Growing manifestation of impatience
5	“We ask you to describe a situation in which you found yourself taking a critical decision alone in the academic/professional context, by taking full responsibility for it and in complete disagreement with the rest of the group.”	Adverse

**Table 2:** Scoring assignment based on the normal distribution of the results obtained during a preliminary pilot test.

Reg_Stress_ Assignment Score—Value (s)		Res_Stress_ Assignment Score—Value (|s|)	
1	≥80	1	≥24
2	60 ÷ 80	2	18 ÷ 24
3	40 ÷ 60	3	12 ÷ 18
4	20 ÷ 40	4	6 ÷ 12
5	≤20	5	≤6

## Data Availability

The data presented in this study are available on request from the corresponding author due to ethical reasons regarding sensitive personal data protection (requests will be evaluated according to the GDPR—Reg. UE 2016/679 and its ethical guidelines).
